# Modulation of Post-Antibiotic Bacterial Community Reassembly and Host Response by *Candida albicans*

**DOI:** 10.1038/srep02191

**Published:** 2013-07-12

**Authors:** John R. Erb Downward, Nicole R. Falkowski, Katie L. Mason, Ryan Muraglia, Gary B. Huffnagle

**Affiliations:** 1 Division of Pulmonary and Critical Care Medicine, Department of Internal Medicine, University of Michigan Medical School

## Abstract

The introduction of *Candida albicans* into cefoperazone-treated mice results in changes in bacterial community reassembly. Our objective was to use high-throughput sequencing to characterize at much greater depth the specific changes in the bacterial microbiome. The colonization of *C. albicans* significantly altered bacterial community reassembly that was evident at multiple taxonomic levels of resolution. There were marked changes in the levels of *Bacteriodetes* and *Lactobacillaceae*. *Lachnospiraceae* and *Ruminococcaceae*, the two most abundant bacterial families, did not change in relative proportions after antibiotics, but there were marked genera-level shifts within these two bacterial families. The microbiome shifts occurred in the absence of overt intestinal inflammation. Overall, these experiments demonstrate that the introduction of a single new microbe in numerically inferior numbers into the bacterial microbiome during a broad community disturbance has the potential to significantly alter the subsequent reassembly of the bacterial community as it recovers from that disturbance.

Exposure to broad spectrum antibiotics, such as cephalosporins, causes marked shifts in bacterial community structure, including decreased numbers, reduced diversity, changes in membership and changes in evenness^1^,^2^,^3^,^4^,^5^. After cessation of antibiotics, bacterial communities in the GI tract will generally reassemble to a pre-antibiotic structure. However, complete recovery is not always the case^1^,^2^,^3^,^4^,^5^. The factors that drive community reassembly have not been completely elucidated. Colonization of the microbiome by a new microbe can occur during the period of diminished colonization resistance that immediately follows antibiotic therapy. This "invasion" of the community has the potential to alter reassembly dynamics through stimulation of mucosal inflammation, alteration of microbial metabolic networks, interference with quorum sensing, and alteration of adhesion^6^,^7^,^8^.

Yeast are naturally resistant to antibacterial antibiotics and the contribution of yeast to modulating post-antibiotic bacterial community reassembly is not well understood. Interplay between yeast and bacteria in polymicrobial communities in the host and environment have been reported^9^,^10^,^11^,^12^, consistent with the concept that growth of yeast populations in the microbiota has the potential to alter bacterial community reassembly. On a healthy mucosa, yeast (e.g., *Candida albicans*) are kept at low levels or excluded by yet-to-be-defined mechanisms of colonization resistance mediated by the indigenous bacterial microbiota^13^,^14^,^15^,^16^,^17^,^18^,^19^. Antibiotics, notably broad spectrum antibiotics, can disrupt colonization resistance, leading to yeast overgrowth (either as an indigenous "bloom" or by exogenous invasion into the disrupted microbiota)^20^,^21^. The cessation of antibiotics can quite often reverse the pathogenic process attributed to high yeast numbers on the mucosa. However, there is an ever-increasing body of research documenting that changes in the bacterial microbiota alone can alter mucosal biology^22^,^23^,^24^,^25^,^26^,^27^,^28^. Thus, there remains the question of whether the bloom or invasion of yeast in a disrupted microbiome can alter bacterial community reassembly following broad disturbance and whether this alteration will modify host mucosal biology.

We have previously addressed this second question about modification of host mucosal biology by yeast. We demonstrated that the presence of *C. albicans* in the GI microbiota during recovery from treatment with a third generation cephalosporin can alter host mucosal immunity at a distal site, rendering mice susceptible to developing allergic airway disease^24^,^25^. Since yeast in the absence of antibiotic disturbance did not render the host susceptible, nor did antibiotics alone, these data raised the possibility that one mechanism may involve alteration of bacterial community reassembly by the introduced yeast. We subsequently demonstrated, using selective culture and 16S rRNA gene clone libraries, that there were changes in bacterial community reassembly when *C. albicans* was present^29^. Our primary objective in this current study was to characterize at much greater depth the specific changes in the bacterial community via 454 pyrosequencing of 16S rRNA gene amplicon libraries, similar to our previous studies analyzing intestinal conventionalization dynamics in mice^30^. Since intestinal inflammation can alter the bacterial microbiota, we also sought to determine whether intestinal inflammation was playing a significant role in this process.

## Results

### Disturbing the indigenous bacterial community by a broad spectrum antibiotic breaks colonization resistance and allows long-term colonization of the ileum and cecum by *C. albicans* CHN1

**Figure 1:**
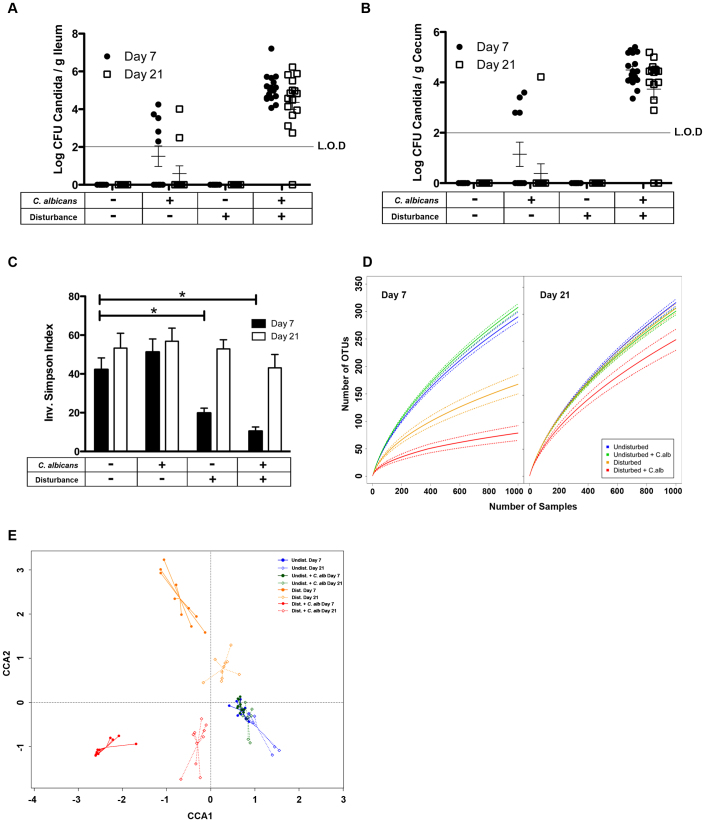
The effect of *C. albicans* colonization on bacterial community structure. C57BL/6 mice were treated with cefoperazone in their drinking water for one week (days -7 to 0), switched to sterile drinking water and then inoculated with *C. albicans* CHN1 by oral gavage at day 0. The groups of mice were then analyzed for bacterial microbiome changes and *C. albicans* colonization at days 7 and 21 (n = 8–12 mice per treatment per time point). Panels A and B demonstrate the growth of *C. albicans* from the ileum and cecum at day 7 and 21 (L.O.D. of 10^1.7^). The inverse-Simpson diversity index was utilized to quantify the diversity of the cecal communities at day 7 and day 21 (panel C). Significance was tested using 1-way ANOVA (p < 0.05). Rarefaction analysis was also employed to view what idealized collector's curve based on the data would look like at day 7 and day 21 (panel D). The mean curve of 8–10 rarefaction curves is displayed as a bold line and the dotted lines represent the standard error. Panel E is a canonical correspondence analysis (CCA) plot of both day 7 and 21 normalized microbial composition data where the data has been constrained by treatment and time. To better visualize the group clusters, each data point is connected to its group centroid.

C57BL/6 mice were treated with cefoperazone in their drinking water for one week (days −7 to 0), then switched to sterile drinking water and inoculated with *C. albicans* CHN1 by oral gavage at day 0. The groups of mice were subsequently analyzed for bacterial microbiome changes and *C. albicans* colonization at days 7 and 21 post-antibiotic treatment (Figs. 1A & 1B). Cefoperazone reduced total bacterial numbers in the intestine by 10,000 fold during the week the mice were drinking the antibiotic water (determined by both culture and total 16S rRNA gene qPCR, data not shown). Upon cessation of the antibiotic, total bacterial numbers returned to pre-antibiotic levels by 72 hours (approx. 10^10^ culturable bacteria per g cecal tissue). Neither untreated mice nor those whose microbiome had been disturbed by cefoperazone had detectable *C. albicans* in their ileum or cecum at days 7 and 21 (Figs. 1A & 1B). When mice were gavaged with *C. albicans* in the absence of microbiome disturbance, less than half of the mice had detectable *C. albicans* levels at day 7 and almost none at day 21. In sharp contrast, all of the gavaged mice with a disturbed microbiome had detectable *C. albicans* colonization at day 7 and the colonization levels did not significantly change through day 21. We have followed mice out through 90 days, and *C. albicans* levels in *C. albicans*-gavaged mice remain similar to those seen at day 21 (data not shown). Thus, the indigenous bacterial microbiota exerts strong competitive exclusion against invasion by the yeast *C. albicans*; however, disturbance of the community by cefoperazone breaks colonization resistance and allows *C. albicans* to colonize the intestinal microbiome at levels far inferior to those of the bacteria (>10^10^ bacteria vs. 10^4^ yeast).

### After disturbance of the GI microbiota, colonization by *C. albicans* CHN1 at low numbers can alter the reassembly of the bacterial community

To assess the effects of *C. albicans* CHN1 colonization on the reassembly of the intestinal bacterial community after broad disturbance, we used 454 pyrosequencing of V3–V5 16S rRNA gene amplicon libraries generated from total cecal mucosal DNA at days 7 and 21 post-disturbance. Sequence data were binned into operational taxonomic units (OTU), based on 97% similarity, as described in the methods.

Two metrics of change in bacterial diversity were calculated for each of the treatment groups at day 7 and day 21: inverse-Simpson values and rarefaction curves (Figs. 1C & 1D). The inoculation of *C. albicans* into an undisturbed community did not significantly affect diversity. However, there was a significant decrease in the diversity of the bacterial community of the cecum at day 7 post-disturbance compared to untreated mice. The inoculation of *C. albicans* into a disturbed community further decreased community diversity at day 7 post-antibiotic, although the additional loss of diversity did not score as statistically significant. By day 21, the diversity of both the groups that had suffered a microbiota disturbance had largely recovered to that of the untreated groups, although the diversity of the group that was disturbed and gavaged with *C. albicans* still trended lower.

Next, the bacterial community structure of the experimental groups was assessed using canonical correspondence analysis (CCA). This is a method of direct gradient analysis commonly used in ecology^37^,^38^ where the community data matrix is subjected to a weighted linear regression on the constraining variables, and a correspondence analysis performed on the fitted values. This both focuses the data on the question of interest and creates a testable model for assessment of significant effects. In our study, the variation was constrained to treatment group and time (Fig. 1E). The communities in the untreated (undisturbed) mice shifted very little over the course of the experiments (day 7 vs. day 21). The addition of *C. albicans* to the undisturbed state had very little effect on the bacterial communities at day 7 or day 21. In contrast, disturbance of the bacterial community by cefoperazone treatment resulted in a significant shift (p < 0.005) away from the untreated state, even seven days after the cessation of antibiotics. The introduction of *C. albicans* into this disturbed bacterial community resulted in a community structure that was not only significantly different from that of untreated mice, but was also significantly different from mice treated with antibiotics alone. Between days 7 and 21 post-antibiotic, the bacterial intestinal communities in both of the disturbed groups changed to become more similar to that in the undisturbed group (i.e., reassembly). However, the bacterial microbiota of mice treated with antibiotics (disturbed) and of antibiotic-disturbed with *C. albicans* gavage remained significantly different from each other.

### Colonization of the ileum and cecum by *C. albicans* elicits a host response but does not induce intestinal inflammation

**Figure 2:**
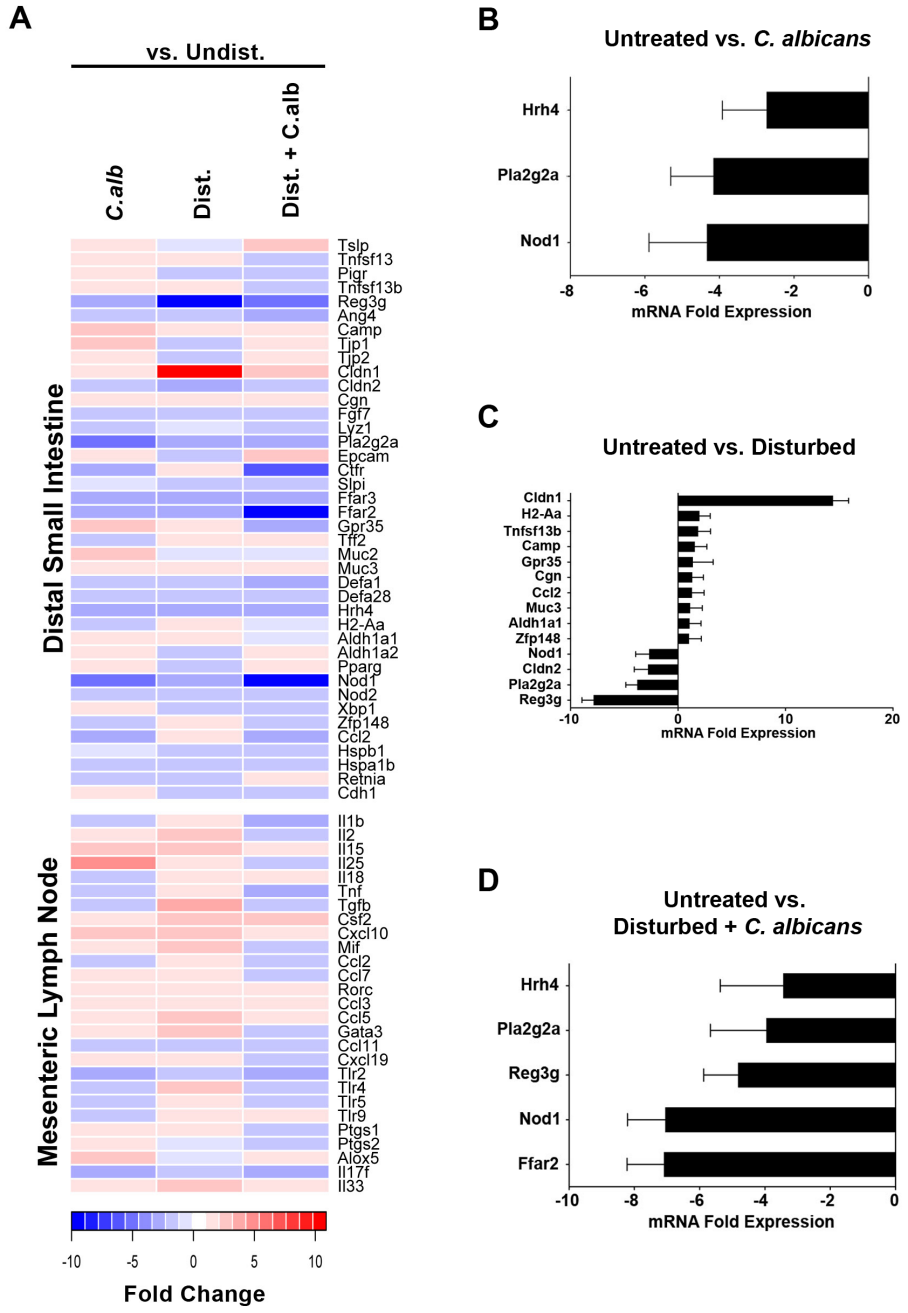
The response of the host to microbiota disturbance and *C. albicans* colonization. (A) Panel A depicts a heatmap of gene expression in the ileum and mesenteric lymph nodes as determined by multiplex qPCR array. For gene identities see Table S1. Fold changes are depicted relative to the undisturbed state. Panels B–D depict genes in which a statistically significant difference occurred are displayed for each of the treatment groups (B) *C. albicans* alone, (C) disturbed microbiota, or (D) disturbed microbiota colonized with *C. albicans*. Significant differences in gene expression were determined using SAM (p < 0.05, false discovery rate set at zero (q = 0.00)) are displayed for each of the treatment groups) as described in Methods.

**Figure 3:**
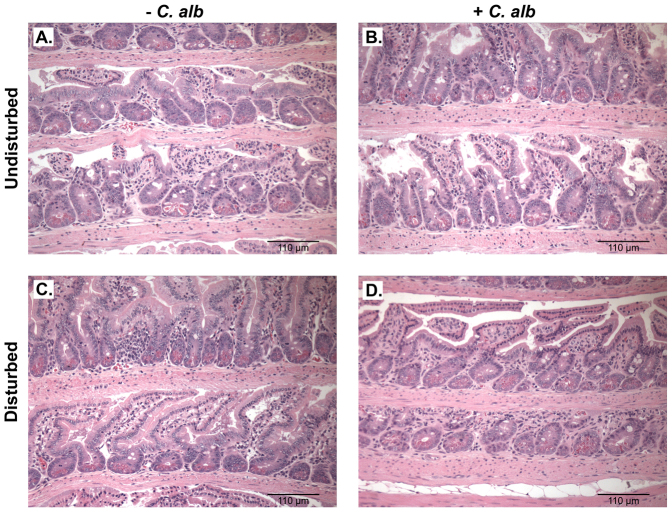
Photomicrograph of the ileum of mice in the treatment groups (Day 21). Figure 3 displays Hematoxylin & Eosin stained histology of the small intestine from mice that were (A) Undisturbed (untreated), (B) *C. albicans* only, (C) disturbed microbiota (Cefoperazone only), or (D) disturbed microbiota colonized with *C. albicans* (disturbed plus *C. albicans*). Mice were treated as described in Fig. 1 and the manuscript text.

Mice were examined at days 7 and 21 post-antibiotic for intestinal inflammation (histology) and at day 21 for changes in intestinal and mesenteric lymph node gene expression by high-throughput qPCR. When *C. albicans* was introduced into an undisturbed microbiota (Fig. 2B), very few genes showed any significant change in expression, while disturbance of the microbiota caused a number of genes to be significantly altered in expression (Fig. 2C). The addition of *C. albicans* to a disturbed microbiota (Fig. 2D) resulted in changes in gene expression not seen in the other groups. The effects of these treatments on the expression of immune modulatory molecules in the mesenteric lymph nodes followed a similar trend (Fig. 2A). However, despite the active differential regulation of genes, there was no evidence of inflammation in histological sections of the small intestine (defined by absence of leukocyte infiltrates, edema or changes in epithelium structure) and no histological evidence of hyphal invasion (Fig. 3). We have reported previously that *C. albicans* colonization does not induce cecal inflammation^25^,^29^.

### Introduction of *C. albicans* into a disturbed bacterial community can alter the diversity and membership of the community

**Figure 4:**
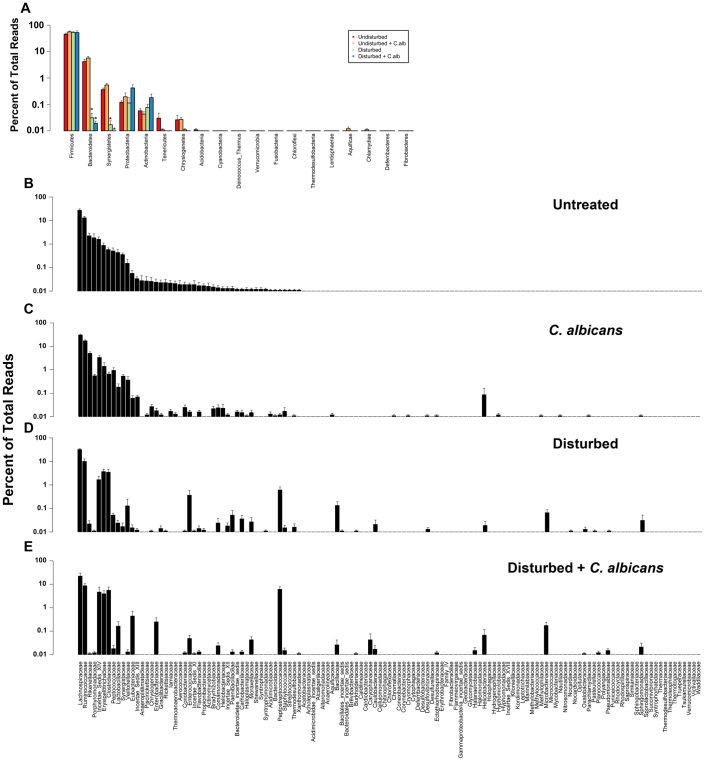
Phylum and family taxonomic membership of day 7 treatment groups. OTUs were classified according to the RDP taxonomy and rank abundance graphs constructed for the phylum and family-level taxonomies. Panel A is the phylum-level classification for the day 7 undisturbed (red), undisturbed plus *C. albicans* (orange), disturbed (green) or disturbed plus *C. albicans* (blue) groups. Panels B–E are the family-level taxonomic distributions. X-axis ordering of the rank abundance graphs is based on the relative distribution in the undisturbed community. Data are mean ± SEM; n = 8–10 mice per group from 3 separate experiments.

We next characterized the bacterial lineages that shifted during disturbance and recovery, by using RDP-based taxonomic assignment of the bacterial OTUs (97% identity), as described in the methods. At day 7, significant phylum-level shifts were evident in both the antibiotic-disturbed community and the community of antibiotic-disturbed with *C. albicans* gavage (Fig. 4A). There were significant decreases in both the *Bacteroidetes* and the *Synergistetes*. The dominant phylum was the *Firmicutes*, which remained unchanged no matter the treatment.

Analysis at a deeper taxonomic level, revealed that there were a number of marked changes in bacterial community membership at the family-level (Figs. 4B–4E). Both the *Rikenellaceae* and the *Porphyromonadaceae*, among the top 5 most abundant families in the undisturbed treatment group, were all but non-existent in either of the disturbed treatment groups. In contrast, bacteria from less abundant families (in untreated mice) bloomed in the mice with the disturbed microbiota.

Furthermore, there were notable changes at day 7 within the *Lachnospiraceae* (Fig. 6A) and *Ruminococcaceae* (Fig. 6B). These two bacterial families were (1) the most abundant in all the experimental groups and (2) did not appear to change in relative proportions after antibiotics. At the genus-level, the disturbance of the microbiota resulted in a marked shift in the dominant genera within these two familes (Fig. 6). In undisturbed communities, *Lachnobacterium*, *Marvinbryantia* and *Syntrophococcus* constituted approximately 80% of the *Lacnospiraceae.* However, after antibiotic treatment, they accounted for <50% of the population at day 7. There were marked blooms of the rare *Lachnospiraceae* genera *Coprococcus*, *Anaerosporobacter*, *Dorea* and *Robinsoniella* in antibiotic-only mice. In contrast, *C. albicans* colonization of the disturbed community was associated with the rapid recovery of *Lachnobacterium* and *Syntrophococcus* to undisturbed levels, outgrowth of *Roseburia*, and *Robinsoniella*, and reduction in *Coprococcus* and *Dorea* levels (Fig. 6). In the *Ruminococcaceae*, disturbance had very little effect on the dominant genera within the community; however, the presence of *C. albicans* in the disturbed community resulted in almost a complete elimination of *Oscillibacter* from the community.

**Figure 5:**
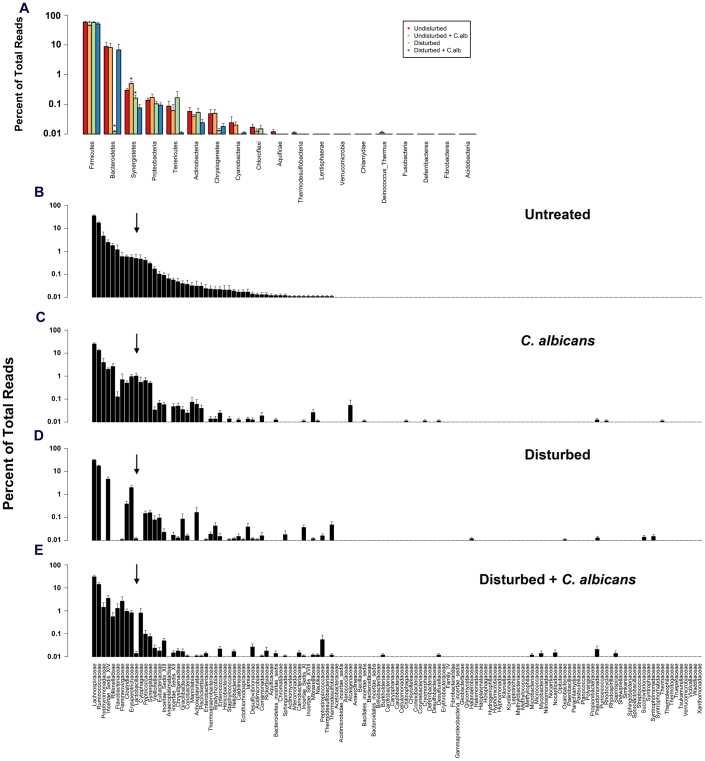
Phylum and family taxonomic membership of day 21 treatment groups. OTUs were classified according to the RDP taxonomy and rank abundance graphs constructed for the phylum and family-level taxonomies. Panel A is the phylum-level classification for the day 21 undisturbed (red), undisturbed plus *C. albicans* (orange), disturbed (green) or disturbed plus *C. albicans* (blue) groups. Panels B–E are the family-level taxonomic distributions. X-axis ordering of the rank abundance graphs is based on the relative distribution in the undisturbed community. The arrows in panels D and E highlight the *Lactobacillaceae*, which remain significantly reduced at day 21 following cefoperazone treatment. Data are mean ± SEM; n = 8–10 mice per group from 3 separate experiments.

**Figure 6:**
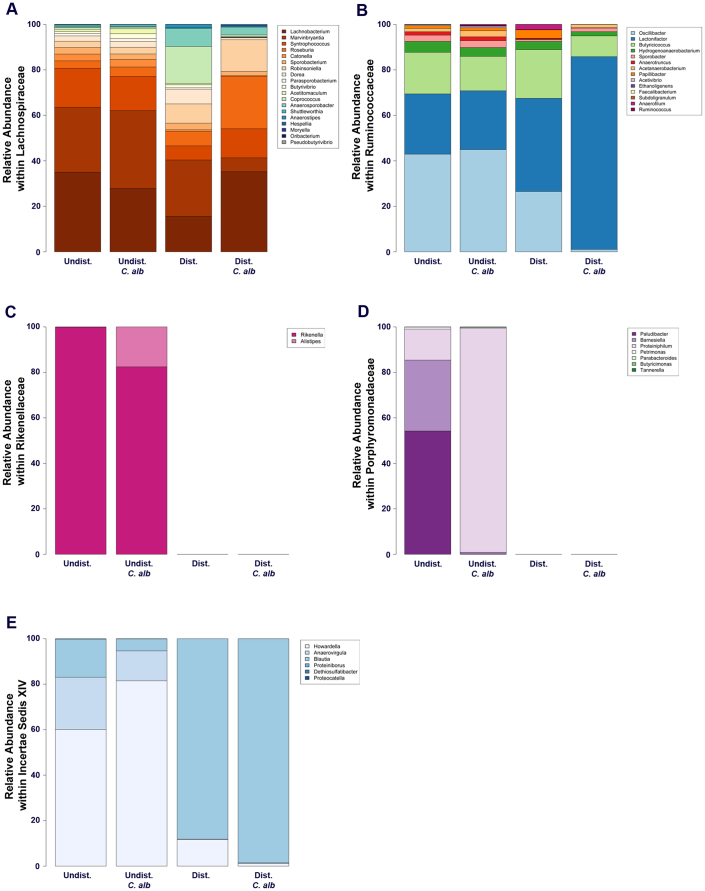
Genus-level taxonomy of the five most abundant families (Day 7). The top 5 most abundant families (*Lachnospiraceae*, *Ruminococcaceae*, *Rikenellaceae*, *Porphyromonadaceae*, and *Incerte Sedis XIV*, respectively) were further classified at a genus-level taxonomy (panels A–E). Data are mean; n = 8–10 mice per group pooled from 3 separate experiments.

**Figure 7:**
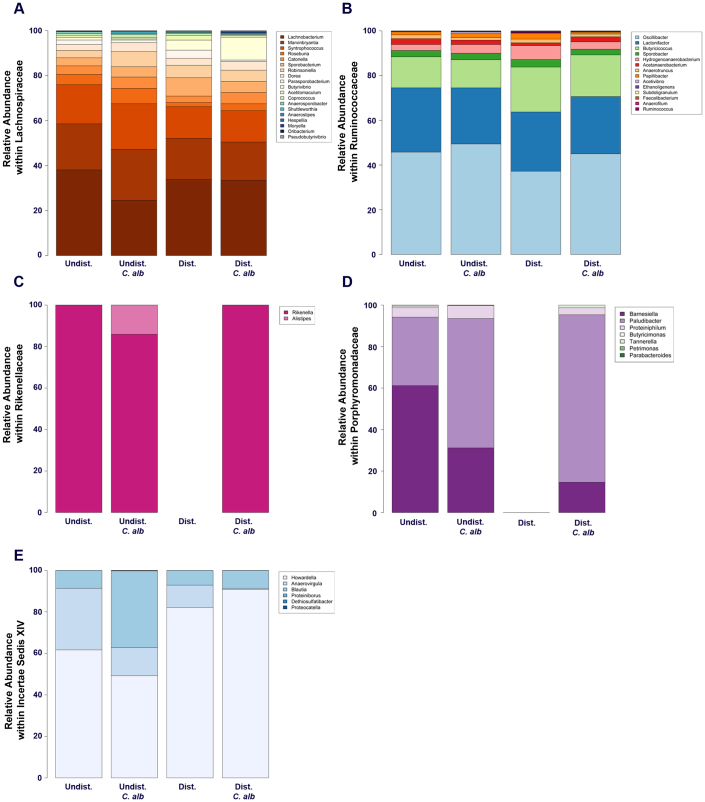
Genus-level taxonomy of the five most abundant families (Day 7). The top 5 most abundant families (*Lachnospiraceae*, *Ruminococcaceae*, *Rikenellaceae*, *Porphyromonadaceae*, and *Incerte Sedis XIV*, respectively) were further classified at a genus-level taxonomy (panels A–E). Data are mean; n = 8–10 mice per group pooled from 3 separate experiments.

A similar analysis was next carried out at the phylum, family and genus-level at day 21 post-disturbance. The bacterial community structure of the antibiotic-treated mice still had not recovered to a pre-antibiotic state (Fig. 5). Most notably, the levels of the *Bacteroidetes* phylum was 100 fold lower level than in undisturbed levels (Fig. 5A). However, the presence of low levels of *C. albicans* within the disturbed community was associated with higher *Bacteroidetes* levels than in antibiotic only treated mice, similar to untreated mice (Fig. 5A). The blooms in low abundance bacteria that were seen at day 7 were no longer evident at day 21. Relative levels of six of the eleven most dominant families were still markedly depressed in antibiotic-only mice at day 21. In contrast, the presence of *C. albicans* during the recovery process was associated with ten of the eleven most dominant bacterial families being found at relative levels similar to that found in untreated (undisturbed) mice. The one notable exception was the very low levels of the *Lactobacillaceae* (denoted by arrows, Figs. 5D & 5E) in both the groups that had undergone an antibiotic-disturbance. In contrast to day 7, there were no longer genus-level differences in the *Lachnospiraceae* and *Ruminococcaceae* at day 21 (Fig. 7), largely indicating that the *Lachnospiraceae* populations had finally recovered fully from cefoperazone treatment.

## Discussion

These experiments demonstrate that the introduction of a single new microbe (the yeast *C. albicans* CHN1) in numerically inferior numbers into the bacterial microbiome during a broad community disturbance can significantly alter the subsequent reassembly of the bacterial community as it recovers from that disturbance. This occurs in the absence of overt intestinal inflammation (leukocyte infiltrates, edema or changes in epithelium structure) but is associated with changes in host gene expression in the intestinal epithelium. We have previously demonstrated that the recovery of the microbiota in the presence of *C. albicans* leads to a state of systemic hyperresponsiveness^24^,^25^. The effects of the antibiotics and yeast were localized to the GI tract because (1) cefoperazone is poorly absorbed when delivered in the drinking water, (2) we have been able to demonstrate that oral treatment with cefoperazone is unable to clear a cefoperazone-sensitive bacteria from the lungs of mice (data not shown), and (3) yeast are not introduced into the lungs following oral gavage^24^,^25^. We propose that our current studies provide additional insight into potential pathways by which colonization with a numerically inferior organism after antibiotics can have a disproportionate effect on the immune system without inducing intestinal inflammation: by affecting the reassembly of the bacterial microbiome.

Our study demonstrates that *C. albicans* can colonize the mouse cecum and ileum without causing overt intestinal inflammation but the presence of *C. albicans* is associated with changes in bacterial community reassembly. Although *C. albicans*, the introduced microbe, is not a normal member of the mouse microbiota, it is known to first colonize humans within a few weeks after birth^39^. In mice, *C. albicans* is excluded by the normal microbiota; however, colonization can take place if the GI microbiota is disturbed. Interestingly, while *C. albicans* causes a mild gastritis in the stomach, the fungi colonizes other parts of the lower GI tract with no evidence of local inflammation^31^. A diverse and abundant fungal microbiome, as detected by molecular techniques has been reported previously, using mice from both a specific pathogen-free (SPF) source and from a colony with a restricted bacterial flora (RF mice)^40^. In a recently published study with Dectin-1 knockout and wildtype mice, high levels of indigenous fungi were reported in wildtype laboratory mice, especially *C. tropicalis* and accounted for 65% of the fungi detected by ITS1-2 amplicon pyrosequencing, although no culture data was presented for this yeast that can be readily cultured on Sabouraud Dextrose Agar and identified on Chromagar^41^. In our laboratory, as we have reported here, we have observed that SPF mice routinely lack culturable fungi. Treatment with a broad spectrum antibiotic, such as cefoperazone, can result in the culture of fungi from intestinal samples on SDA, such as *C. tropicalis*^25^,^29^,^31^, but after cessation of cefoperazone, these yeast are rarely detected in the mice. The systemic hyperresponsiveness of *Candida*-colonized mice to challenge with an aeroallergen^24^,^25^ indicates that intestinal *C. albicans* colonization can alter the immune response of the host. The recent report demonstrating a role for *Candida* species in driving colitis in Dectin-1 knockout and wildtype mice^41^ further underscores the potential of the fungal microbiome to alter host immunity and inflammation.

The current work also highlights that members of the *Porphyromonadaceae*, *Rikenellaceae*, *Lachnospiraceae* and *Lactobacillaceae* bacterial families are some of the most susceptible to perturbations in reassembly following cefoperazone treatment (+/− *C. albicans*). While it is unknown how the mouse microbiota prevents *C. albicans* colonization, previous studies have demonstrated the ability of *C. albicans* to compete with bacteria (particularly amongst members of the *Firmicutes*)^42^. Interactions between *C. albicans* and other phyla, such as the *Bacteroidetes*, are not well investigated, although it has been suggested that *Lactobacillus* species are critical^15^,^43^. To induce a disturbance in the microbial community and enable *C. albicans* colonization, cefoperazone was used. This is a poorly absorbed third-generation cephalosporin with excellent activity against anaerobic bacteria^44^. It can facilitate gastric colonization by *C. albicans*^31^. Cefoperazone has also been shown to be able to disturb the mouse cecal microbiota for greater than six weeks after washout if the mice are housed in isolation^1^. This delay in reassembly is in contrast to the observation that a cecal microbiota community can completely reassemble within seven days following transfer from a conventional to a germ-free mouse, despite a complete destruction of community structure during the first few days after transfer^30^. In addition, cefoperazone-treated mice will completely and quickly reassemble their cecal microbiota if allowed to recover in the presence of an untreated mouse^1^. While currently of unknown significance, the association between *C. albicans* colonization and altered *Lachnospiraceae* composition is intriguing in light of the report that *Lachnospiraceae* communities can be important in colonization resistance against enteric bacterial pathogens^45^.

We have observed that the intestinal microbiota shift caused by the introduction of *C. albicans* into cefoperazone-treated mice is not associated with mucosal inflammation, but it is also not a neutral event for the host. The pattern of genes down-regulated in mice with a disturbed microbiota and *C. albicans* colonization is consistent with an environment where the host is less responsive to microbial signals. Down-regulated genes of interest included Ffar2 (Gpr43)^46^,^47^,^48^, Nod1^49^,^50^, Gpr35^51^,^52^, and the TLRs^26^,^53^. These are all well-characterized as environmental sensors that play important roles in inflammation. At the same time, there is a decrease in the expression of various anti-microbial peptides and tight-junction proteins that might render the host less capable of maintaining microbial homeostasis^54^,^55^,^56^. Thus, it is possible that the communication between the host and the GI microbiota has been disrupted but not to the breaking point to create intestinal inflammation. It also remains possible that, despite a lack of intestinal inflammation, the mild inflammation in the stomach may be influencing gene expression in the cecum, although it is unclear how this signaling would take place. Another possibility is that formation of discrete foci of yeast invasion and inflammation is occurring in the intestine but is undetectable at the level of resolution used in this study. Finally, active suppression of host responsiveness by *C. albicans* or other members of the microbiota remains a possibility because it has been previously demonstrated that *C. albicans* can produce prostaglandins and related oxylipins^57^,^58^,^59^. In summary, we have provided a proof-of-concept study that low-level colonization and/or blooms of yeast within the microbiome following antibiotic therapy can potentially have unintended consequences on bacterial microbiota reassembly and host mucosal immunity.

## Methods

### Animals and housing

Female C57BL/6 mice (Jackson Laboratories, Indianapolis, IN) were housed 5 mice to a cage under specific-pathogen-free conditions in enclosed filter-top cages. The experiments (at days 7 and 21 post-treatment) were performed on 3 separate occasions. Food and sterile water were given *ad libitum*. Food remained constant throughout the experiment to minimize the effect of diet on the microbiota. Mice were maintained on grates by the Unit for Laboratory Animal Medicine (ULAM) at the University of Michigan (Ann Arbor, MI) to prevent coprophagy. All protocols were approved by the University of Michigan University Committee on Use and Care of Animals (UCUCA).

### Antibiotic treatment

Cefoperazone (0.5 mg/ml) (Sigma-Aldrich, St. Louis, MO) was administered orally to mice *ad libitum* in drinking water. Antibiotic treatment was continued for 7 days (days −7 to day 0) prior to *C. albicans* colonization. After 7 days, antibiotic containing drinking water was replaced with sterile water.

### *C. albicans* GI inoculation

*C. albicans* strain CHN1 (a human clinical isolate) was grown as previously described^31^, diluted to 2 × 10^8^ CFU/ml in sterile nonpyrogenic saline, and 10^7^ CFU in 50 μl gavaged using a 24-gauge feeding needle attached to a 1-ml syringe mounted on a Stepper repetitive pipette (Tridak, Brookfield, CT) to deliver an equivalent amount of inoculum to each mouse. The inoculums were plated on Sabouraud dextrose agar (SDA) to verify the number of colony forming units (CFU) delivered.

### Tissue isolation

Following euthanasia, the GI tract was sterilely removed and the cecum separated from the colon and small intestine. A 1 cm^2^ tissue strip from the cecal tip was removed, rinsed in sterile 1× PBS and divided for DNA and RNA isolation. The remaining portion of the cecum was cut open along the greater curvature and rinsed in sterile 1× PBS. Thin slices along the outer portion were removed and fixed overnight in 10% Neutral Buffered Formalin for histological analysis. The remaining tissue was homogenized in sterile water using a Tissuemiser (Fisher Scientific). Aliquots of the homogenates were plated in 10-fold serial dilutions on Sabouraud dextrose agar (Difco). For the small intestine, the distal half of the organ was separated, cut open longitudinally and rinsed in sterile 1× PBS. A 1 cm^2^ tissue strip was taken from the middle and divided for DNA and RNA isolation. The distal portion of the remaining tissue was prepared for histological analysis using the "Swiss-roll" technique whereby the tissue is rolled and fixed overnight in 10% Neutral Buffered Formalin, which more readily allows visualization of the entire length of the intestinal sample. The proximal portion of the remaining tissue was homogenized in sterile water using a Tissuemiser (Fisher Scientific). Aliquots of the homogenates were plated in 10-fold serial dilutions on Sabouraud dextrose agar (Difco) to determine levels of *C. albicans* colonization.

### DNA extraction

Genomic DNA was extracted from tissue homogenized in 360 μl ATL buffer (Qiagen DNeasy Blood & Tissue kit) using a modified protocol previously demonstrated to isolate bacterial DNA^31^.

### 454-prosequencing

The V3–V5 region of the bacterial 16S rRNA gene was targeted using barcoded primer sets corresponding to 357F and 929R. These primers sets were originally developed by the Broad Institute. The PCR conditions were 95°C for 2 min., followed by 30 cycles of (95°C 20 sec, 50°C 30 sec, 72°C 5 min), holding at 4°C. Quality control and sequencing was carried out at the University of Michigan, using the Roche 454 GS-FLX Titanium platform.

### 16S rRNA gene data analysis

Sequence data were processed and analyzed using the software *mothur* v. 1.21^32^ according to the protocol provided (http://www.mothur.org). The operational taxonomic units (OTUs) were binned at 97% similarity (to create shared community file and the phylotyped, genus-level grouping, file). For the purposes of this study, we focused on OTUs that were present at greater than 0.5% of the sample population. These files, along with the file containing the taxonomic information for the OTUs, were imported and further analyzed in R. The R-package vegan^33^ was used for diversity analyses and ordinations, as well as a custom R script for sorting classification results into tables (ClassifierSorter v.2). Classification of OTUs was carried out using the implementation of the RDP Classifier and RDP taxonomy training set 7^34^,^35^ contained in *mothur*. The number of sequences in each sample group can be found in Supplemental Table S2.

### Quantification of mRNA levels using high-throughput quantitative PCR

RNA was isolated from each tissue using TRIzol (Ambion) and the RNeasy Mini Kit (Qiagen). RNA concentration was determined using a Nanodrop instrument (Thermo Scientific). Complementary DNA (cDNA) was generated using the RT^2^ First Strand Kit (Qiagen). Expression levels of genes under study were determined using Mouse RT^2^Profiler PCR assays, custom-made to contain replicate sets of 48 primer pairs (Qiagen). Each well of the replicate sets was loaded with 2.5 ng of cDNA reaction product. Each card was run on a LightCycler 480 Real-Time PCR system (Roche). The relative RNA expression levels were inferred from the *C_t_* values. The geometric mean of ß-actin and GAPDH levels was used as the internal control for the generation of ΔCt values. The heatmap was generated in R using the package gplots^36^.

### Statistics

A standard one-way ANOVA with a Tukey post-test was used to determine whether statistical differences existed in *C. albicans* CFU counts and in diversity metrics (p < 0.05 considered significant). The R implementation of the Statistical Analysis of Microarrays (SAM) (R-package samr) was used to determine statistical differences in gene expression with significance (p < 0.05) being set for q = 0.00 (q-value of a gene equals the minimum estimated false discovery rate at which it appears significant). Differences in microbial community centroids were determined using a permutational MANOVA (function adonis() in the R-package vegan). The significance of constraints in constrained ordinations was tested using an ANOVA-like permutation test (function anova.cca in R-package vegan). All statistical analyses were performed in R or Prism v5.

## Author Contributions

Designed project: J.E.D., K.M. and G.H. Performed experiments: K.M., N.F. and J.E.D. Analyzed Data: J.E.D., G.H., J.E.D. and R.M. Generated Figures: J.E.D., G.H. and R.M. Wrote the manuscript: J.E.D. and G.H. All authors reviewed the manuscript.

## Supplementary Material

Supplementary Information Supplementary
