# Supplementary material for: Modulation of Post-Antibiotic Bacterial Community Reassembly and Host Response by Candida albicans
Source: Sci Rep. 2013 Jul 12;3:2191. doi: 10.1038/srep02191 (PMC3709164; doi:10.1038/srep02191)
Supplement: Supplementary Information — Supplementary [file srep02191-s1.pdf]

Modulation of Post-Antibiotic Bacterial Community Reassembly and Host Response by *Candida albicans*

John R. Erb Downward, Nicole R. Falkowski, Katie L. Mason, Ryan Muraglia, and Gary B. Huffnagle

Table S1

Gene ID	Description
Tslp	Thymic astomal lymphopoietin
Tnfsf13	Tumor necrosis factor (ligand) superfamily, member 13
Pigr	Polymeric immunoglobulin receptor
Tnfsf13b	Tumor necrosis factor (ligand) superfamily, member 13b
Reg3g	Regenerating islet-derived 3 gamma
Ang4	Angiogenin, ribonuclease A family, member 4
Camp	Cathelicidin antimicrobial peptide
Tjp1	Tight junction protein 1
Tjp2	Tight junction protein 2
Cldn1	Claudin 1
Cldn2	Claudin 2
Cgn	Cingulin
Fgf7	Fibroblast growth factor 7
Lyz1	Lysizyme 1
Pla2g2a	Phospholipase A2, group IIA (platelets, synovial fluid)
Epcam	Epithelial cell adhesion molecule
Ctfr	Cystic fibrosis transmembrane conductance regulator homolog
Slpi	Secretory leukocyte peptidase inhibitor
Ffar3	Free fatty acid receptor 3
Ffar2	Free fatty acid receptor 2
Gpr35	G protein-coupled receptor 35
Tff2	Trefoil factor 2 (spasmolytic protein 1)
Muc2	Mucin 2
Muc3	Mucin 3, intestinal
Defa1	Defensin, alpha 1
Defa28	Defensin, alpha 28
Defb1	Defensin beta 1
Defb3	Defensin beta 3
Hrh4	Histamine receptor H4
H2-Ea	Histocompatibility 2, class II antigen E alpha
H2-Aa	Histocompatibility 2, class II antigen A alpha
Aldh1a1	Aldehyde dehydrogenase family 1 subfamily A1
Aldh1a2	Aldehyde dehydrogenase family 1 subfamily A2
Pparg	Peroxisome proliferator activated receptor gamma
Nod1	Nucleotide-binding oligomerization domain containing 1
Nod2	Nucleotide-binding oligomerization domain containing 2
Xbp1	X-box binding protein 1
Shh	Sonic hedgehog
Zfp148	Zinc finger protein 148
Tcf4	Transcription factor 4
CCL2	Chemokine (C-C Motif) ligand 2
Hspb1	Heat shock protein 1
Hspa1b	Heat shock protein 1B

Table S1 (continued)

Retnla	Resistin like alpha
Cdh1	Cadherin 1
IL-1b	Interlukin 1- beta
IL-2	Interlukin 2
IL-15	Interlukin 15
IL-25	Interlukin 25
IL-18	Interlukin 18
TNF-alpha	Tumor necrois factor alpha
TGF-beta	Transforming growth factor beta
CSF-2	Colony stimulating factor 2 (GM-CSF)
CXCL10	chemokine (C-X-C motif) ligand 10
MIF	macrophage migration inhibitory factor
CCL2	chemokine (C-C motif) ligand 2
CCL7	chemokine (C-C motif) ligand 7
Rorc	RAR-related orphan receptor C
CCL3	chemokine (C-C motif) ligand 3
CCL5	chemokine (C-C motif) ligand 5
Gata3	GATA binding protein 3
CCL11	chemokine (C-C motif) ligand 11
CXCL19	chemokine (C-X-C motif) ligand 19
TLR2	Toll-like receptor 2
TLR4	Toll-like receptor 4
TLR5	Toll-like receptor 5
TLR9	Toll-like receptor 9
Ptgs1	Prostaglandin endoperoxide synthase 1
Ptgs2	Prostaglandin endoperoxide synthase 2
Alox5	arachidonate 5-lipoxygenase
IL-17f	Interlukin 17f
IL-33	Interlukin 33

Modulation of Post-Antibiotic Bacterial Community Reassembly and Host Response by *Candida albicans*

John R. Erb Downward, Nicole R. Falkowski, Katie L. Mason, Ryan Muraglia, and Gary B. Huffnagle

Table S2

Day 7 Read Distribution

Group	# Reads	S.E.M	n
Unt	22884	243.38	10
C.alb	27117	221.27	10
Disturbed	36208	360.95	10
Disturbed + C. alb	44587	629.15	10

Day 21 Read Distribution

Group	# Reads	S.E.M	
Unt	29729	245.9	10
C.alb	18300	219.45	8
Disturbed	24208	202.21	10
Disturbed + C. alb	31354	296.19	10

Modulation of Post-Antibiotic Bacterial Community Reassembly and Host Response by *Candida albicans*
John R. Erb Downward, Nicole R. Falkowski, Katie L. Mason, Ryan Muraglia, and Gary B. Huffnagle

Supplemental Figures

Supplemental Table S1 - List of gene identifiers and common names of genes analyzed for Figure 2A.

Supplemental Table S2 - a breakdown of the number of reads obtained for each treatment group for each time point.
